# Supplementary material for: A Controlled Trial in Community Pediatrics to Empower Parents Who Are at Risk for Parenting Stress: The Supportive Parenting Intervention
Source: Int J Environ Res Public Health. 2019 Nov 15;16(22):4508. doi: 10.3390/ijerph16224508 (PMC6888243; doi:10.3390/ijerph16224508)
Supplement: Supplementary file 1 [file ijerph-16-04508-s001.pdf]

International Journal of
Environmental Research and Public Health

MDPI

Supplementary Materials

Supplementary material 1

Description of the IPARAN

The IPARAN is designed to identify risk factors related to the development of parenting stress. These risk factors were identified by Bouwmeester-Landweer et al. (22) based on previous studies (8-19) and can be divided into three general domains based on the ecological model of Belsky (24-26) and the concept of parental awareness by Newberger (27), which was elaborated upon by Baartman (28). These three domains are: *i*) child and family characteristics; *ii*) parental developmental history and personality (including parental awareness); and *iii*) characteristics of the social network.

The IPARAN is a three-page self-report form that has a general part (filled in by both the mother and father), a part for the mother to complete, and a part for the father (or other parent) to complete (see Table S1). The general part consists of five items that assess the following risk factors in the first domain (child and family characteristics): the child's birth weight, duration of the pregnancy, the age of the father when the child was born, the age of the mother when the child was born, and the family structure (child lives with both biological parents or does not live with both biological parents).

The parts completed by the mother and father (or other parent) consist of sixteen items each. These items assess risk factors in the all three domains. The item in the first domain is "quarrels with partner ever become physical". The items in the second domain include "worried about raising your child", "unhappy during pregnancy about becoming a mother/father", "parents or caregivers had a loving relationship", "ever hit as a child", "felt unhappy in the past 3 years", "losing temper", "negative sexual experience", "drug/alcohol abuse", and "hitting should be part of upbringing". The items in the third domain include "finding it difficult to ask for help", "feeling comfortable in the neighborhood", "maintain close relations with family", "receive support from network of family, neighbors, and friends", and "receive support from partner". The items in the three domains are answered using either a 4-point response scale (always, often, sometimes, or never) or a yes/no option.

Each risk factor is assigned a score ranging from 0 to 2 (see S1 Table). To categorize a parent as being at risk or not at risk for parenting problems, a total score is calculated for each parent by adding the parent's score to the score from the general part of the instrument. If either parent has a total score of ≥3, the family is considered to be at risk for developing parenting problems.

Table S1. Score assigned if a parent meets the criteria for risk factors on the IPARAN.

**General risk factors**	**Score**	
Child's birth weight <2500 grams	1.0	
Gestational age <38 weeks	0.5	
Single parent family structure	2.0	
Age of the mother *<19 years*	0.5 (18 years) or 1.0 (<18 years)	
Age of the father *<19 years*	0.5 (18 years) or 1.0 (<18 years)	
**Father and mother part**	**Score of the Father**	**Score of the Mother**
Worried about raising your child *(always or often)*	1.0	2.0
Unhappy during pregnancy about becoming a mother/father *(always or often)*	1.0	1.0
Parents (or caregivers) had a loving relationship *(sometimes or never)*	1.0	1.0
Felt safe with parents (or caregivers) as a child *(sometimes or never)*	1.0	1.0
Were hit as a child *(always, often, or sometimes)*	1.0	1.0
Hitting should be part of upbringing *(yes)*	1.0	1.5
Should use less alcohol or drugs *(yes)*	0.5	0.5
Ever had a negative sexual experience *(yes)*	1.0	1.5

International Journal of Environmental Research and Public Health
MDPI

Felt unhappy in the past 3 years *(always or often)*	1.0	2.0
Losing temper *(always-often)*	1.0	1.0
Find it difficult to ask for help *(always or often)*	NA[1]	1.0
Quarrels with partner become physical *(always, often, or sometimes)*	2.0	1.0
Feel comfortable in the neighborhood *(sometimes or never)*	NA[1]	0.5
Maintain close relations with family *(sometimes or never)*	NA[1]	0.5
Receive support from network of family, neighbors, and friends *(sometimes or never)*	NA[1]	0.5
Receive support from partner *(sometimes or never)*	0.5	1.5

[1] NA, not applicable, as these questions are not part of the summation score for the father.

Table S2. Results from the linear regression model evaluating the association between research condition and CBCL scores.

	**Model 1[1]**		**Model 2[2]**	
	**Intervention group vs control group**		**Intervention group vs control group**	
	**Beta coefficient (95% CI)**	***p*-value[3]**	**Beta coefficient (95% CI)**	***p*-value[3]**
**CBCL Total problem score**	6.42 (0.89; 11.95)	0.023	1.49 (-4.58; 7.56)	0.630
**CBCL Internal problem score**	4.90 (0.21; 3.99)	0.030	0.70 (-1.41; 2.80)	0.517
**CBCL External problem score**	1.94 (-0.06; 3.93)	0.057	0.45 (-1.76; 2.65)	0.691

Note: 95% CI, 95% Confidence Interval
[1] Model without correction for confounders.
[2] Model corrected for age of the child, nationality of the mother, income and family structure.
[3] *p*-values were calculated using a generalized linear model.

Table S3. Interaction analyses.

	**Competence as a parent**		**CBCL Total problem**		**CBCL Internal problem**		**CBCL External problem**	
	**Beta coefficient *(95% CI)***	***p*–value[1]**	**Beta coefficient *(95% CI)***	***p*–value[1]**	**Beta coefficient *(95% CI)***	***p*–value[1]**	**Beta coefficient *(95% CI)***	***p*–value[1]**
**Group*Income**			15.01 (2.47; 27.55)	0.019	6.82 (2.43; 11.20)	0.002		
**Group*Family structure**	0.95 (0.13; 1.77)	0.023			-6.20 (-12.11; -0.29)	0.040		
**Group*IPARAN score**							-0.85 (-1.50; -0.20)	0.011

Notes: Only significant ($p<0.05$) results of the interaction analyses are presented; 95% CI, 95% Confidence Interval;
[1] *p*-values were calculated using a generalized linear model.
